# Lactate dehydrogenase-to-albumin ratio predicts 30-day and 90-day mortality in glucocorticoid-treated ICU patients with pneumonia: a secondary analysis of a multicenter cohort

**DOI:** 10.3389/fmed.2026.1832943

**Published:** 2026-05-05

**Authors:** Dandan Han, Shengfa Sun, Ai Liu, Cuiping Hao, Jianping Sun

**Affiliations:** Department of Critical Care Medicine, Affiliated Hospital of Jining Medical University, Jining, Shandong, China

**Keywords:** glucocorticoids, intensive care unit, lactate dehydrogenase-to-albumin ratio, mortality, pneumonia, risk stratification

## Abstract

**Background:**

Glucocorticoid therapy in pneumonia patients confounds conventional inflammatory biomarkers, hindering accurate risk stratification. The lactate dehydrogenase-to-albumin ratio (LAR), a proposed steroid-resilient index reflecting cellular injury and nutritional reserve, may offer prognostic utility in this population.

**Methods:**

This secondary analysis of a multicenter cohort included 499 ICU patients with pneumonia receiving glucocorticoids. The optimal LAR cutoff for 30-day mortality was determined via the Youden index. Associations with 30- and 90-day mortality were assessed using multivariable Cox regression and propensity score matching (PSM), with doubly robust estimation serving as the primary analysis in the matched cohort. Incremental predictive value was evaluated using the net reclassification improvement (NRI) and integrated discrimination improvement (IDI).

**Results:**

The optimal LAR cutoff was 13.39. High LAR (≥13.39) was associated with significantly higher 30-day (45.8% vs. 12.1%) and 90-day (51.4% vs. 14.3%) mortality (both *p* < 0.001). After full multivariable adjustment, high LAR remained an independent predictor for 30-day (HR 2.07, 95% CI 1.34–3.20) and 90-day (HR 1.93, 95% CI 1.29–2.89) mortality. In the PSM cohort, the doubly robust estimate yielded consistent findings (30-day HR 2.07, 95% CI 1.34–3.21; 90-day HR 1.93, 95% CI 1.29–2.90). Sensitivity analyses treating LAR as a continuous variable and as tertiles confirmed a dose–response relationship independent of any single threshold. LAR demonstrated good discrimination (AUC ≈ 0.74) and provided significant incremental prognostic value when added to the PSI score (NRI 0.299, *p* = 0.048).

**Conclusion:**

LAR is a simple, readily available, and steroid-resilient biomarker that independently predicts short- and mid-term mortality in pneumonia patients receiving glucocorticoids. Incorporating LAR into clinical assessment may enhance early risk stratification and guide individualized management in this challenging population.

## Introduction

Pneumonia remains one of the most common causes of morbidity and mortality worldwide, contributing significantly to intensive care unit (ICU) admissions and posing a major public health challenge (1). Despite advances in antimicrobial therapy and supportive care, adjunctive glucocorticoid therapy has become increasingly utilized in severe cases to mitigate the exaggerated inflammatory response and potentially improve clinical outcomes (2, 3). However, the administration of exogenous steroids introduces notable alterations in the immune and metabolic landscape, complicating clinical management and decision-making (4).

One of the primary consequences of glucocorticoid use is its interference with traditional biomarkers of inflammation and infection. Existing evidence suggests that pneumonia patients on similar glucocorticoid regimens can exhibit substantially divergent clinical trajectories (5). Addressing this variability necessitates a precision-medicine approach, wherein accurate individual risk stratification plays a pivotal role in optimizing therapy and improving survival outcomes (6, 7). Consequently, there is a pressing need for reliable biomarkers that remain informative even in the presence of steroid therapy.

Conventional inflammatory markers, such as C-reactive protein (CRP) and procalcitonin (PCT), have long been recognized for their prognostic value in various infectious diseases (8, 9). However, in patients receiving glucocorticoids, these biomarkers are often confounded by steroid-induced immunosuppression and reduced hepatic synthesis, leading to an underestimation of ongoing tissue injury and physiological deterioration (9, 10). Specifically, glucocorticoids suppress acute-phase protein production, often attenuating the CRP response even in the presence of substantial lung damage (10).

In contrast, certain biomarkers appear less susceptible to such pharmacologic interference. Serum albumin (ALB), which reflects nutritional status, oncotic pressure, and overall physiological reserve, has been consistently linked to clinical outcomes in pneumonia, with hypoalbuminemia correlating with increased mortality and prolonged ICU stays (11–13). Similarly, lactate dehydrogenase (LDH) is a sensitive, albeit non-specific, marker of tissue hypoxia and irreversible cellular injury. In the context of respiratory infections, elevated LDH levels correlate with the extent of lung parenchymal damage and serve as early indicators of acute respiratory distress syndrome (ARDS) and clinical deterioration (14, 15). Furthermore, composite indices such as the blood urea nitrogen-to-albumin ratio (BUN/ALB) have also demonstrated prognostic value in pneumonia, reflecting the integrated status of acute prerenal injury, heightened catabolism, and nutritional depletion (16, 17). Nevertheless, each of these individual markers provides only a partial view of the underlying pathophysiology, limiting their independent prognostic utility.

The lactate dehydrogenase-to-albumin ratio (LAR) is a composite index that integrates cellular injury and nutritional reserve. By combining LDH, which reflects irreversible cellular damage, with albumin, which indicates overall physiological reserve and capillary leakage, LAR may offer a more comprehensive assessment of disease burden. Moreover, since LDH release is minimally affected by the direct pharmacological actions of glucocorticoids, this ratio may possess unique “steroid-resilient” characteristics (10). In recent years, LAR has demonstrated promise as a prognostic tool across several critical conditions, including sepsis, COVID-19, and pulmonary embolism (18–20).

To our knowledge, no large-scale studies have specifically evaluated the prognostic value of LAR in pneumonia patients receiving glucocorticoid therapy. Therefore, utilizing a multicenter retrospective cohort and robust statistical methods, this study aims to assess the ability of admission LAR to predict 30-day and 90-day all-cause mortality in this high-risk population. Furthermore, we sought to evaluate the incremental predictive value of LAR beyond established clinical scoring systems and to examine its applicability across various clinical subgroups, with the ultimate goal of establishing a simple, readily available, and steroid-resilient tool for individualized survival prediction in the ICU setting.

## Methods

### Study design and population

This study represents a *post hoc* secondary analysis of a multicenter retrospective cohort derived from a publicly available dataset hosted in the Dryad repository. The original dataset was collected by Li et al. across six secondary and tertiary teaching hospitals in China between January 1, 2013 and December 31, 2017. The parent study was approved by the Ethics Committee of the China-Japan Friendship Hospital (Approval No. 201586) and was released under the CC BY-NC 4.0 license. Since the present analysis utilized fully de-identified data, it was exempt from additional ethical review and was conducted in accordance with the Strengthening the Reporting of Observational Studies in Epidemiology (STROBE) guidelines (21).

The parent cohort included 716 ICU patients who received either oral or intravenous glucocorticoids for underlying conditions such as connective tissue diseases, chronic glomerulonephritis, or nephrotic syndrome. “Glucocorticoid accumulation” in the original dataset is defined as the standardized daily dose (in methylprednisolone equivalent), not the cumulative total dose over the entire treatment period. This measure has been explicitly defined for clarity. All enrolled patients received oral or intravenous prednisone ≥20 mg/day (or equivalent) for at least 14 days according to the inclusion criteria of the original study by Li et al. (22). Pneumonia was defined according to the American Thoracic Society/Infectious Diseases Society of America (ATS/IDSA) guidelines (23), requiring new pulmonary infiltrates on imaging, along with at least one of the following: new or worsening respiratory symptoms, fever or hypothermia, signs of lung consolidation, or abnormal white blood cell count.

**Figure 1:**
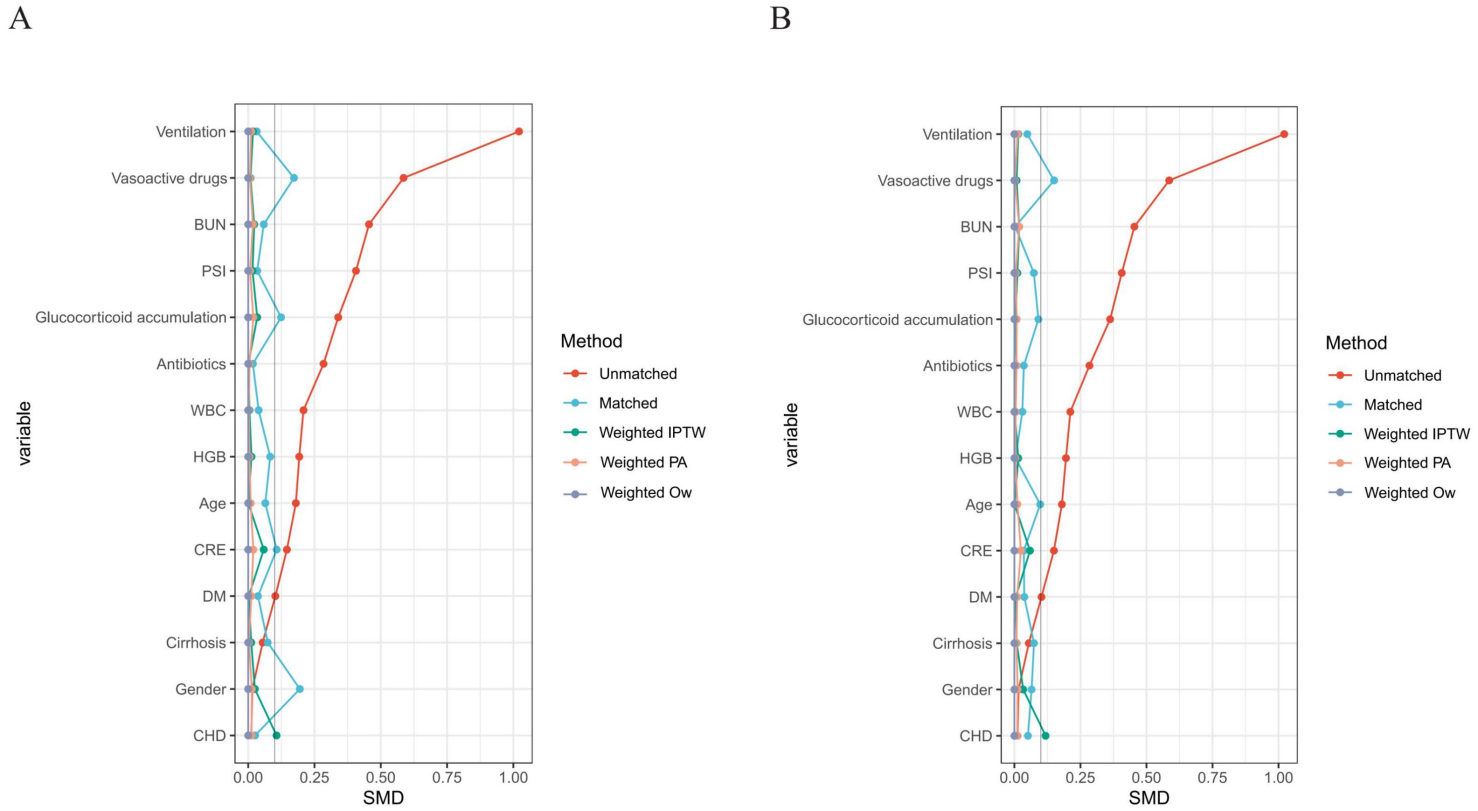
Assessment of covariate balance via Love plot for the investigation of 30-day **(A)** and 90-day **(B)** mortality. The vertical dashed line at SMD = 0.1 represents the threshold for adequate covariate balance. Values within ±0.1 indicate negligible standardized differences between groups. BUN, blood urea nitrogen; CHD, coronary heart disease; CRE, creatinine; DM, diabetes mellitus; HGB, hemoglobin; PSI, pneumonia severity index; WBC, white blood cell count; IPTW, inverse probability of treatment weighting; OW, overlap weighting; SMD, standardized mean difference.

Inclusion criteria for this analysis were as follows: (1) age ≥ 16 years, (2) receipt of systemic glucocorticoid therapy, and (3) pneumonia diagnosed at ICU admission or new-onset pneumonia during hospitalization that necessitated ICU care. Exclusion criteria included: (1) non-infectious pulmonary lesions (e.g., malignancy-associated infiltrates or non-infectious interstitial lung disease) and (2) missing laboratory values required for the calculation of the lactate dehydrogenase-to-albumin ratio (LAR). Following exclusions (203 patients lacking LDH data and 14 lacking albumin data), 499 patients remained for analysis (Figure 1).

### Exposure, outcomes, and covariate definitions

The primary exposure was LAR, calculated as the ratio of lactate dehydrogenase (LDH) (U/L) to albumin (g/L) (20).

The primary outcomes were all-cause mortality at 30 days and 90 days following ICU admission. The optimal LAR cut-off value for predicting 30-day mortality was determined using the maximally selected Youden index derived from the receiver operating characteristic (ROC) curve within this cohort (24). Patients were subsequently categorized into a low-LAR group (< 13.39) and a high-LAR group (≥ 13.39). To minimize potential bias associated with the data-driven cut-off, the following sensitivity analyses were performed: (1) LAR was modeled as a continuous variable (per 1-unit increase) in Cox regression models, and (2) trend testing was conducted across LAR tertiles (24, 25).

Covariates were selected based on clinical relevance and prior literature (22) and included: demographics (age, sex), comorbidities (coronary heart disease, diabetes mellitus, liver cirrhosis), disease severity scores (Pneumonia Severity Index [PSI], CURB-65) (26), treatment-related factors (mechanical ventilation, vasoactive drug use, antibiotic therapy, cumulative glucocorticoid dose) (27, 28), and laboratory values (hemoglobin, white blood cell count, creatinine, blood urea nitrogen).

### Management of missing data

Data for LDH and albumin were complete; however, other covariates exhibited varying levels of missingness (Supplementary Table S1). Missing values were addressed using multiple imputation by chained equations (MICE) (29). Five imputed datasets were generated, incorporating the exposure, outcomes, and all covariates; results were pooled using Rubin’s rules (29). A complete-case analysis was conducted as a sensitivity check.

### Statistical analysis

Continuous variables were reported as mean ± standard deviation or median (interquartile range), depending on their distribution. Categorical variables were presented as frequencies and percentages. Group comparisons were performed using Student’s t-test, Wilcoxon rank-sum test, chi-square test, or Fisher’s exact test, as appropriate.

### Propensity score-based analyses

To account for confounding, propensity scores were calculated using multivariable logistic regression to predict membership in the high-LAR group based on all baseline covariates. Propensity score matching (PSM) was performed using a 1:1 nearest-neighbor matching approach with a caliper of 0.2 times the standard deviation of the logit of the propensity score (24). Separate matched cohorts were generated for the 30-day mortality (*n* = 248) and 90-day mortality (*n* = 246) analyses. Covariate balance was assessed using standardized mean differences (SMD), with an SMD < 0.1 considered indicative of acceptable balance, and was visualized using Love plots. Robustness checks included inverse probability of treatment weighting (IPTW), Propensity Score Adjustment(PA), and overlap weighting (OW).

### Survival analyses

Kaplan–Meier curves and log-rank tests were used to compare survival between groups in both the full and matched cohorts (24). Cox proportional hazards models were employed to estimate hazard ratios (HRs) and 95% confidence intervals (CIs) for the association between LAR and mortality. Three nested models were constructed for the unmatched cohort: Model 1 (adjusted for age and sex), Model 2 (additionally adjusted for comorbidities), and Model 3 (further adjusted for laboratory values, treatments, glucocorticoid dose, and PSI score) (24, 28). In the matched cohort, an additional Model 4 was constructed, which was identical to Model 3 except that the PSI score was replaced with the CURB-65 score, to verify whether the independent prognostic value of LAR was robust to the specific disease severity score employed for adjustment. The association was evaluated across nine analytical frameworks, including crude analysis, multivariable analysis, doubly robust estimation, propensity score adjustment, PSM, IPTW, and OW (24). The proportional hazards assumption was tested using Schoenfeld residuals and was confirmed in all primary analyses.

### Predictive performance and incremental value assessment

The discriminative ability of LAR, compared with LDH, albumin, PSI, CURB-65, and other albumin-based ratios (NE/ALB, PLT/ALB, BUN/ALB, CRP/ALB), was assessed using ROC curves. Areas under the curve (AUCs) with 95% CIs were compared using the DeLong test (26, 30). The incremental prognostic value of LAR beyond established scoring systems was quantified using the continuous net reclassification improvement (NRI) and integrated discrimination improvement (IDI) (24). The following comparisons were performed: PSI + LAR vs. PSI alone, CURB-65 + LAR vs. CURB-65 alone, and LAR vs. LDH alone (31).

### Subgroup and interaction analyses

The consistency of the association was assessed in prespecified subgroups, including age (< 60 vs. ≥ 60 years), sex, CURB-65 score (≤ 1 vs. > 1), high-dose glucocorticoid use, mechanical ventilation, and diabetes status (28, 32). Analyses were repeated in both the full and matched cohorts. Effect modification was examined by including multiplicative interaction terms (LAR continuous × subgroup variable) in Cox models.

### Sensitivity analyses

To ensure the robustness of our findings, we integrated and conducted a series of sensitivity analyses, specifically including: (1) to overcome potential overfitting bias associated with the data-driven cut-off, LAR was modeled as a continuous variable (per 1-unit increase) and as tertile categories in multivariable Cox regression analyses within the unmatched original cohort (n = 499), to verify the presence of a dose–response relationship independent of any specific threshold; (2) the primary multivariable Cox regression analysis using the same binary cut-off (LAR ≥ 13.39 vs. < 13.39) was repeated in the unmatched original cohort, to assess the consistency of effect estimates before and after matching; (3) unadjusted Kaplan–Meier survival curves were plotted for the original cohort; (4) prespecified subgroup analyses were performed in the original cohort; and (5) a complete-case analysis was conducted to evaluate the impact of missing data handling strategies. The results of all aforementioned sensitivity analyses are presented in detail in the main text or the Supplementary materials (24, 28).

### Software and statistical significance

All statistical analyses were performed using R software (version 4.2.2; R Foundation for Statistical Computing, Vienna, Austria) and the Free Statistics platform (version 2.3). Statistical tests were two-sided, and a *p* value < 0.05 was considered statistically significant.

## Results

### Baseline characteristics and propensity score matching

A total of 499 ICU patients with pneumonia receiving glucocorticoid therapy were included in the final analysis. Using the optimal LAR cut-off of 13.39 derived from the ROC-Youden index, patients were categorized into a low-LAR group (< 13.39, *n* = 322, 64.5%) and a high-LAR group (≥ 13.39, *n* = 177, 35.5%) (Figure 1, Supplementary Figure S1, Supplementary Table S2).

Before matching, the high-LAR group exhibited greater disease severity, with higher rates of mechanical ventilation (65.5% vs. 20.5%), vasoactive drug use (31.6% vs. 9.0%), and PSI scores (88.5 ± 32.8 vs. 75.8 ± 29.5; all *p* < 0.001) (Table 1). Laboratory differences included lower hemoglobin (110.1 ± 21.9 vs. 114.5 ± 23.5 g/L, *p* = 0.042) and higher blood urea nitrogen (7.1 vs. 5.7 mmol/L, *p* < 0.001) in the high-LAR group, and antibiotic use was more frequent (76.3% vs. 63.4%, *p* = 0.003). Correspondingly, 30-day mortality was significantly higher in the high-LAR group (45.8% vs. 12.1%, *p* < 0.001) (Supplementary Table S3).

**Table 1:** Imbalance of patient characteristics before and after propensity score matching in the assessment of 30-day mortality.

Variables	Primary cohort			SMD- 0.1	PSM cohort			SMD -0.1
	Low-LAR ratio *n* = 322 (64.5%)	High-LAR ratio *n* = 177 (35.5%)	SMD		Low-LAR ratio *n* = 124 (50%)	High-LAR ratio *n* = 124 (50%)	SMD	
Age^c^, years ≥60	178 (55.3)	82 (46.3)	0.18	>0.1	63 (50.8)	59 (47.6)	0.065	<0.1
Female^c^	159 (49.4)	86 (48.6)	0.016	<0.1	53 (42.7)	65 (52.4)	0.195	>0.1
Ventilation^c^	66 (20.5)	116 (65.5)	1.022	>0.1	61 (49.2)	63 (50.8)	0.032	<0.1
PSI^a^, points	75.8 ± 29.5	88.5 ± 32.8	0.406	>0.1	83.6 ± 33.4	82.5 ± 31.3	0.034	<0.1
Vasoactive drugs^c^	29 (9)	56 (31.6)	0.586	>0.1	25 (20.2)	17 (13.7)	0.173	>0.1
Glucocorticoid accumulation^b^, mg	5.4 (2.5, 12.3)	3.3 (1.9, 5.8)	0.34	>0.1	4.1 (2.4, 7.8)	4.1 (2.2, 6.8)	0.124	>0.1
HGB^a^, g/L	114.5 ± 23.5	110.1 ± 21.9	0.193	>0.1	113.2 ± 21.9	111.4 ± 21.6	0.083	<0.1
BUN^b^, mmol/L	5.7 (4.3, 8.1)	7.1 (5.3, 12.0)	0.456	>0.1	6.0 (4.4, 9.6)	6.3 (5.0, 9.8)	0.059	<0.1
CHD^c^	37 (11.5)	21 (11.9)	0.012	<0.1	14 (11.3)	13 (10.5)	0.026	<0.1
Cirrhosis^c^	2 (0.6)	2 (1.1)	0.055	<0.1	2 (1.6)	1 (0.8)	0.074	<0.1
DM^c^	87 (27)	40 (22.6)	0.102	>0.1	33 (26.6)	31 (25)	0.037	<0.1
WBC^a^, ×10^9^/L	7.5 (5.5, 10.7)	8.9 (6.2, 12.1)	0.209	>0.1	7.6 (5.5, 11.1)	8.5 (6.2, 11.9)	0.039	<0.1
Antibiotics^c^	204 (63.4)	135 (76.3)	0.284	>0.1	88 (71)	87 (70.2)	0.018	<0.1
CRE^b^, μmol/L	63.2 (51.3, 82.2)	65.0 (48.3, 105.0)	0.146	>0.1	61.8 (49.2, 88.5)	60.5 (46.6, 89.3)	0.107	>0.1

An absolute standardized mean difference (SMD) < 0.1 is considered indicative of acceptable covariate balance. PSI, pneumonia severity index; HGB, hemoglobin; BUN, blood urea nitrogen; CHD, coronary heart disease; DM, diabetes mellitus; WBC, white blood cell count; LAR, lactate dehydrogenase to albumin ratio; CRE, creatinine; SD, standard deviation; IQR, interquartile range.

a Data presented are mean ± SD.

b median (IQR).

c N (%).

Similar patterns were observed for 90-day mortality (Table 2 and Supplementary Tables S4), with higher rates of mechanical ventilation, vasoactive drug use, and PSI scores in the high-LAR group (all *p* < 0.001). Notably, the cumulative daily glucocorticoid dose was significantly lower in the high-LAR group compared with the low-LAR group (3.2 vs. 5.3 mg, *p* < 0.001). This observation suggests that mortality was primarily driven by the severity of infection and organ injury rather than solely by steroid exposure. Possible explanations include: (1) patients in the high-LAR group were more critically ill, prompting clinicians to cautiously limit glucocorticoid dosage due to concerns regarding secondary infections and other complications (i.e., confounding by indication); and (2) this group may represent a clinical phenotype that is relatively less responsive to glucocorticoid therapy. The 90-day mortality rate was also higher in the high-LAR group (51.4% vs. 14.3%, *p* < 0.001).

**Table 2:** Imbalance of patient characteristics before and after propensity score matching in the assessment of 90-day mortality.

Variables	Primary cohort			SMD- 0.1	PSM cohort			SMD -0.1
	Low-LAR ratio *n* = 322 (64.5%)	High-LAR ratio *n* = 177 (35.5%)	SMD		Low-LAR ratio *n* = 123(50%)	High-LAR ratio *n* = 123(50%)	SMD	
Age^c^, years ≥60	178 (55.3)	82 (46.3)	0.18	>0.1	64 (52)	58 (47.2)	0.098	<0.1
Female^c^	159 (49.4)	86 (48.6)	0.016	<0.1	61 (49.6)	65 (52.8)	0.065	<0.1
Ventilation^c^	66 (20.5)	116 (65.5)	1.022	>0.1	59 (48)	62 (50.4)	0.049	<0.1
PSI^a^, points	75.8 ± 29.5	88.5 ± 32.8	0.406	>0.1	84.8 ± 34.8	82.4 ± 31.4	0.074	<0.1
Vasoactive drugs^c^	29 (9)	56 (31.6)	0.586	>0.1	25 (20.3)	18 (14.6)	0.15	>0.1
Glucocorticoid accumulation^b^, mg	5.3 (2.4, 11.9)	3.2 (1.9, 5.5)	0.362	>0.1	3.6 (2.0, 6.9)	3.7 (2.2, 6.2)	0.091	<0.1
HGB^a^, g/L	114.6 ± 23.5	110.1 ± 21.9	0.195	>0.1	111.5 ± 25.3	111.4 ± 21.6	0.004	<0.1
BUN^b^, mmol/L	5.7 (4.3, 8.1)	7.1 (5.3, 12.0)	0.454	>0.1	6.6 (4.7, 10.7)	6.3 (5.0, 9.8)	0.003	<0.1
CHD^c^	37 (11.5)	21 (11.9)	0.012	<0.1	15 (12.2)	13 (10.6)	0.051	<0.1
Cirrhosis^c^	2 (0.6)	2 (1.1)	0.055	<0.1	2 (1.6)	1 (0.8)	0.074	<0.1
DM^c^	87 (27)	40 (22.6)	0.102	>0.1	32 (26)	30 (24.4)	0.037	<0.1
WBC^a^, ×10^9^/L	7.5 (5.5, 10.7)	8.9 (6.2, 12.1)	0.212	>0.1	7.8 (5.8, 11.3)	8.6 (6.2, 11.9)	0.03	<0.1
Antibiotics^c^	204 (63.4)	135 (76.3)	0.284	>0.1	88 (71.5)	86 (69.9)	0.036	<0.1
CRE^b^, μmol/L	63.2 (51.3, 82.2)	66.9 (48.3, 105.0)	0.15	>0.1	64.0 (51.8, 93.7)	61.9 (46.6, 89.6)	0.036	<0.1

An absolute standardized mean difference (SMD) < 0.1 is considered indicative of acceptable covariate balance. PSI, pneumonia severity index; HGB, hemoglobin; BUN, blood urea nitrogen; CHD, coronary heart disease; DM, diabetes mellitus; WBC, white blood cell count; LAR,lactate dehydrogenase to albumin ratio; CRE, creatinine; SD, standard deviation; IQR, interquartile range.

a Data presented are mean ± SD.

b Median (IQR).

c N (%).

After 1:1 nearest-neighbor propensity score matching, covariate balance improved substantially overall, as reflected by the post-matching SMD columns in Tables 1, 2, although several variables remained mildly imbalanced. In the matched 30-day cohort (*n* = 248), mortality was 37.1% in the high-LAR group versus 26.6% in the low-LAR group (Supplementary Table S3). In the matched 90-day cohort (*n* = 246), corresponding mortality was 40.7% versus 29.3% (Supplementary Table S4). Although the direct comparison of absolute mortality rates between groups did not reach the conventional threshold of statistical significance after matching (*p* = 0.076 and *p* = 0.061, respectively), the direction of effect remained consistent, and subsequent survival analyses that accounted for time-to-event relationships and covariate adjustment provided clear statistical support for the association.

### Association between LAR and mortality

High LAR was consistently associated with increased 30-day and 90-day mortality across multiple analytical frameworks (Table 3). In crude pre-matching analyses, the high-LAR group had a 4.90-fold higher risk of 30-day mortality (95% CI: 3.34–7.19, *p* < 0.001) and a 4.82-fold higher risk of 90-day mortality (95% CI: 3.38–6.87, *p* < 0.001). After full multivariable adjustment, these associations remained statistically significant: HR = 2.07 (95% CI: 1.34–3.20, *p* = 0.001) for 30-day mortality and HR = 1.93 (95% CI: 1.29–2.89, *p* = 0.001) for 90-day mortality (Table 3).

**Table 3:** Associations between LAR and the outcome in the crude analysis, multivariable analysis, and propensity-score analyses.

Analysis	30-day mortality		90-day mortality	
	HR (95% CI)	*P* value	HR (95% CI)	*P* value
Doubly robust with all covariates	2.07 (1.34–3.21)	0.001	1.93 (1.29–2.90)	0.001
Doubly robust with unbalanced covariates	1.86 (1.19–2.90)	0.007	1.70 (1.12–2.57)	0.013
With matched	1.59 (1.01–2.48)	0.043	1.59 (1.04–2.44)	0.033
Crude analysis*	4.90 (3.34–7.19)	<0.001	4.82 (3.38–6.87)	<0.001
Multivariable analysis*	2.07 (1.34–3.20)	0.001	1.93 (1.29–2.89)	0.001
Adjusted for Propensity Score	1.82 (1.17–2.82)	0.008	1.67 (1.11–2.51)	0.014
With inverse probability weighting	1.70 (1.18–2.44)	0.027	1.64 (1.17–2.30)	0.03
With PA	1.69 (1.07–2.69)	0.012	1.58 (1.03–2.42)	0.018
With Ow	1.67 (0.94–2.95)	0.016	1.56 (0.92–2.65)	0.023

The ‘With matched’ row represents the crude (unadjusted) hazard ratio in the propensity score-matched cohort. For stepwise multivariable adjustment within the PSM cohort, see Table 4.*Sensitivity analysis. Multivariable analysis (pre-matching cohort, *n* = 499): adjust for Age, Sex, CHD, DM, Cirrhosis, HGB, WBC, CRE, BUN, Ventilation, Vasoactive drugs, PSI, Glucocorticoid accumulation, Antibiotics. Doubly robust with unbalanced covariates:adjust for Sex, Vasoactive drugs, Glucocorticoid accumulation, CRE. Doubly robust with all covariates:adjust for Age, Sex, CHD, DM, Cirrhosis, HGB, WBC, CRE, BUN, Ventilation, Vasoactive drugs, PSI, Glucocorticoid accumulation, Antibiotics. BUN, blood urea nitrogen; CHD, coronary heart disease; CRE, creatinine; DM, diabetes mellitus; HGB, hemoglobin; PSI, pneumonia severity index; WBC, white blood cell count; PA, propensity-adjusted weighting; OW, overlap weighting.

**Figure 2:**
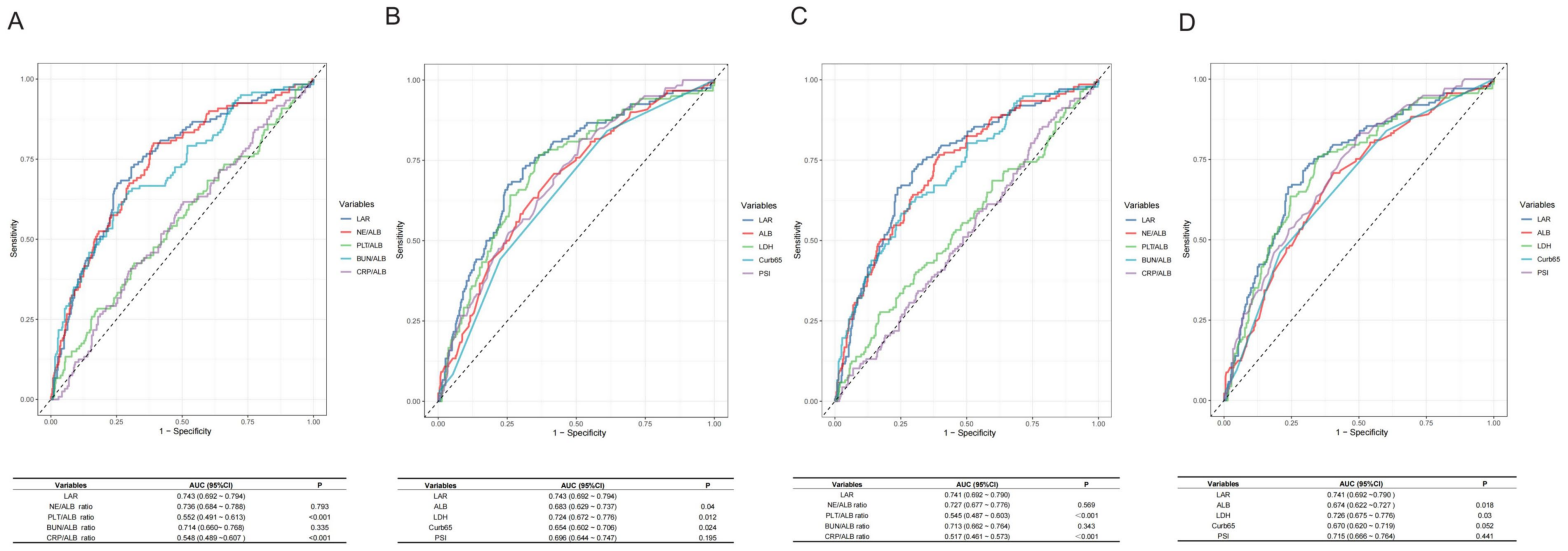
Receiver operating characteristic (ROC) curves of LAR and other clinical indicators for predicting 30-day and 90-day mortality. **(A,B)** 30-day mortality prediction: **Panel A** compares LAR with other ratios (NE/ALB, PLT/ALB, BUN/ALB, and CRP/ALB); **Panel B** compares LAR with individual components (ALB, LDH) and established clinical scores (CURB-65, PSI). **(C,D)** 90-day mortality prediction: **Panel C** compares LAR with other ratios (NE/ALB, PLT/ALB, BUN/ALB, and CRP/ALB); **Panel D** compares LAR with individual components (ALB, LDH) and established clinical scores (CURB-65, PSI). ROC, receiver operating characteristic; AUC, area under the curve; CI, confidence interval; LAR, lactate dehydrogenase-to-albumin ratio; NE%/ALB, neutrophil percentage-to-albumin ratio (neutrophil%/albumin in g/L); PLT/ALB, platelet-to-albumin ratio; BUN/ALB, blood urea nitrogen-to-albumin ratio; CRP/ALB, C-reactive protein-to-albumin ratio; ALB, albumin; LDH, lactate dehydrogenase; Curb65, Confusion, Urea, Respiratory rate, Blood pressure and age ≥65 score; PSI, pneumonia severity index.

In the propensity score-matched cohort, we designated the doubly robust estimate (adjusted for all 14 covariates) as the primary analysis. This analysis demonstrated that high LAR remained independently associated with 30-day mortality (HR = 2.07, 95% CI: 1.34–3.21, *p* = 0.001) and 90-day mortality (HR = 1.93, 95% CI: 1.29–2.90, *p* = 0.001). In contrast, the unadjusted HR in the matched cohort was 1.59 (*p* = 0.043); this discrepancy reflects the presence of residual confounding after matching, which was effectively addressed by the doubly robust approach. Sensitivity analyses using IPTW and overlap weighting yielded comparable HRs (1.56–1.70, all *p* < 0.05), further confirming the robustness of the association (Figure 2).

Stepwise multivariable Cox regression in the matched cohort further strengthened these findings: in Model 3, which adjusted for demographics, comorbidities, laboratory values, and PSI score, high LAR was associated with an HR of 2.14 for 30-day mortality (95% CI: 1.32–3.46, *p* = 0.002) and an HR of 2.05 for 90-day mortality (95% CI: 1.30–3.22, *p* = 0.002) (Table 4). Notably, in Model 4, which replaced the PSI score with the CURB-65 score, the prognostic value of LAR remained highly significant and the effect estimates remained stable (30-day HR = 2.52, 95% CI: 1.51–4.21, *p* < 0.001; 90-day HR = 2.55, 95% CI: 1.59–4.08, *p* < 0.001), indicating that the independent predictive ability of LAR is robust to the specific disease severity score employed for adjustment. These results collectively confirm LAR as an independent prognostic predictor beyond demographics, comorbidities, laboratory values, and disease severity scores.

**Table 4:** Stepwise multivariable Cox regression analysis in the propensity score-matched cohort.

Variable		Crude model		Model 1		Model 2		Model 3		Model 4	
		HR (95% CI)	*P* value	HR (95% CI)	*P* value	HR (95% CI)	*P* value	HR (95% CI)	*P* value	HR (95% CI)	*P* value
30-mortality	LAR < 13.39	1 (Ref)		1 (Ref)		1 (Ref)		1 (Ref)		1 (Ref)	
	LAR ≥ 13.39	1.59 (1.01–2.48)	0.043	1.58 (1.01–2.47)	0.045	1.57 (1.00–2.45)	0.05	2.14 (1.32–3.46)	0.002	2.52 (1.51 ~ 4.21)	<0.001
90-mortality	LAR < 13.39	1 (Ref)		1 (Ref)		1 (Ref)		1 (Ref)		1 (Ref)	
	LAR ≥ 13.39	1.59 (1.04–2.44)	0.033	1.63 (1.06–2.51)	0.025	1.61 (1.05–2.48)	0.029	2.05 (1.30–3.22)	0.002	2.55 (1.59 ~ 4.08)	<0.001

Model 1: adjust for Age, Sex; Model 2: adjust for model 1 + CHD, DM and Cirrhosis; Model 3: adjust for model 2 + HGB, WBC, CRE, BUN, Ventilation, Vasoactivedrugs, PSI, Glucocorticoidaccumulation, Antibiotics; Model 4: adjust for model 2 + HGB, WBC, CRE, BUN, Ventilation, Vasoactivedrugs, CURB-65, Glucocorticoidaccumulation, Antibiotics; BUN, blood urea nitrogen; CHD, coronary heart disease; CRE, creatinine; DM, diabetes mellitus; HGB, hemoglobin; PSI, pneumonia severity index; WBC, white blood cell count; LAR,lactate dehydrogenase to albumin ratio; CURB-65, Confusion, Urea, Respiratory rate, Blood pressure, and Age ≥ 65 years score.

### Predictive performance and incremental value of LAR

**Figure 3:**
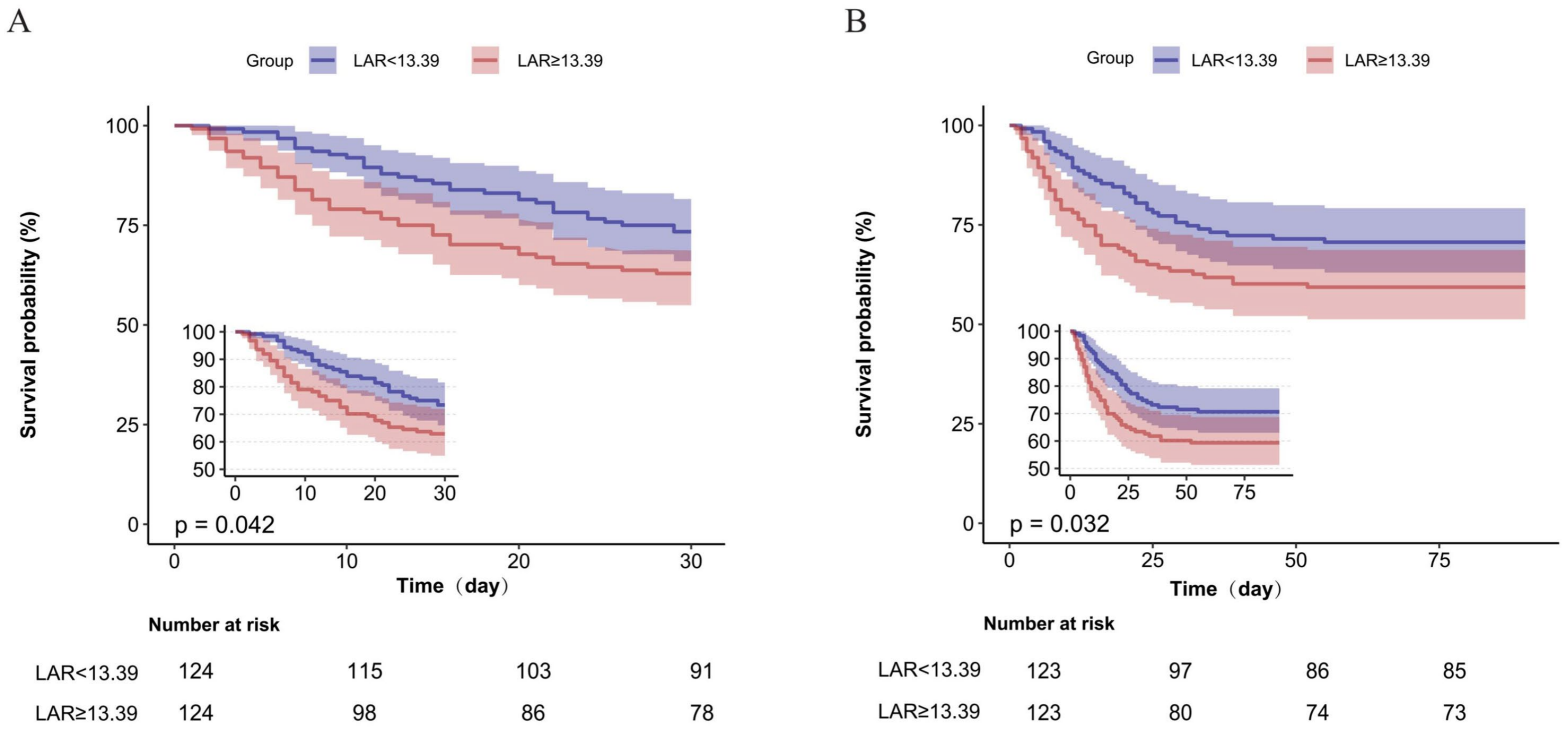
Kaplan–Meier survival analysis by LAR status in the propensity score-matched cohort. **(A)** 30-day and **(B)** 90-day survival curves comparing Low-LAR (<13.39, *n* = 124, blue) versus High-LAR (≥13.39, *n* = 124, red) groups after propensity score matching. Insets magnify early survival divergence. Log-rank test: *p* = 0.042 (30-day) and *p* = 0.032 (90-day), indicating significant survival differences. The “Number at risk” tables show patient counts at key follow-up intervals. LAR, lactate dehydrogenase-to-albumin ratio; PSM, propensity score matching.

LAR demonstrated good discriminative ability for both endpoints. ROC analysis yielded an AUC of 0.743 (95% CI: 0.692–0.794) for 30-day mortality and 0.741 (95% CI: 0.692–0.790) for 90-day mortality (Figure 3). For 30-day prediction, LAR outperformed LDH (AUC 0.743 vs. 0.682, *p* = 0.012) and CURB-65 (0.743 vs. 0.654, *p* = 0.024), while performing comparably to PSI (0.743 vs. 0.710, *p* = 0.195). Other albumin-based ratios (PLT/ALB, CRP/ALB) were also inferior to LAR (all *p* < 0.001). For 90-day mortality, LAR maintained superiority over LDH (*p* = 0.030) and ALB (*p* = 0.018), with a borderline statistically significant advantage over CURB-65 (*p* = 0.052).

Reclassification analyses indicated added prognostic value for LAR (Table 5). Incorporating LAR into the PSI score yielded a continuous NRI of 0.299 for 30-day mortality (95% CI: 0.000–0.403, *p* = 0.048), correctly reclassifying 29.9% of patients. The addition of LAR to the CURB-65 score showed a directionally positive improvement, although it did not reach statistical significance. LAR also significantly improved IDI compared with LDH alone (0.019, 95% CI: 0.001–0.052, *p* = 0.040), demonstrating enhanced risk stratification capability.

**Table 5:** Reclassification and discrimination improvement of LAR compared with clinical scores (PSI, CURB-65) and single biomarker (LDH) for 30-day and 90-day mortality.

Comparison	30-day mortality			90-day mortality		
	Estimate	95% CI	*p*-value	Estimate	95% CI	*P*-value
Model 1: PSI + LAR vs. PSI						
NRI (continuous)	**0.299**	(0.000, 0.403)	**0.048**	0.281	(−0.073, 0.390)	0.072
IDI	0.020	(−0.002, 0.046)	0.092	0.018	(−0.002, 0.042)	0.100
Model 2: CURB-65 + LAR vs. CURB-65						
NRI (continuous)	0.253	(−0.324, 0.376)	0.172	0.240	(−0.387, 0.355)	0.220
IDI	0.013	(−0.006, 0.037)	0.180	0.011	(−0.007, 0.032)	0.232
Model 3: LAR vs. LDH						
IDI	**0.019**	(0.001, 0.052)	**0.040**	0.016	(−0.001, 0.045)	0.064
NRI (continuous)	0.035	(−0.128, 0.216)	0.531	0.025	(−0.159, 0.193)	0.647

Model 1 evaluates the incremental predictive value of adding LAR to the PSI score; Model 2 evaluates the incremental predictive value of adding LAR to the CURB-65 score; Model 3 compares the predictive performance of LAR versus LDH alone. NRI (continuous) and IDI were employed to quantify the improvement in risk reclassification and integrated discrimination, respectively. *P* < 0.05 denotes statistical significance. CI, confidence interval; CURB-65, Confusion, Urea, Respiratory rate, Blood pressure, and Age ≥ 65 years score; IDI, integrated discrimination improvement; LAR, lactate dehydrogenase-to-albumin ratio; LDH, lactate dehydrogenase; NRI, net reclassification improvement; PSI, pneumonia severity index. Bold values indicate statistically significant results.

### Kaplan–Meier and subgroup analyses

**Figure 4:**
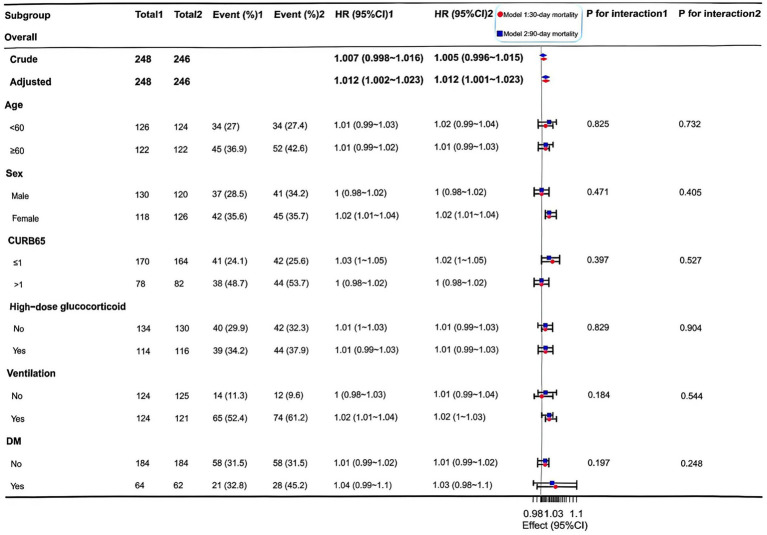
Forest plot of hazard ratios for lar-associated mortality risk stratified by Clinical subgroups in the propensity score-matched cohort. Forest plot showing HRs (95% CIs) for 30-day (pink circles) and 90-day (blue squares) mortality per 1-unit increase in lactate dehydrogenase-to-albumin ratio (LAR) across clinical subgroups in the PSM cohort (*n* = 248). Stratification by age, sex, CURB-65 score, glucocorticoid use, mechanical ventilation, and diabetes mellitus. The vertical dashed line represents HR = 1.0 (null effect). Most subgroups showed consistent associations (P for interaction >0.05). CI, confidence interval; CURB-65, Confusion, Urea, Respiratory rate, Blood pressure, and Age ≥ 65 years score; DM, diabetes mellitus; HR, hazard ratio; LAR, lactate dehydrogenase-to-albumin ratio; PSM, propensity score matching.

**Figure 5:**
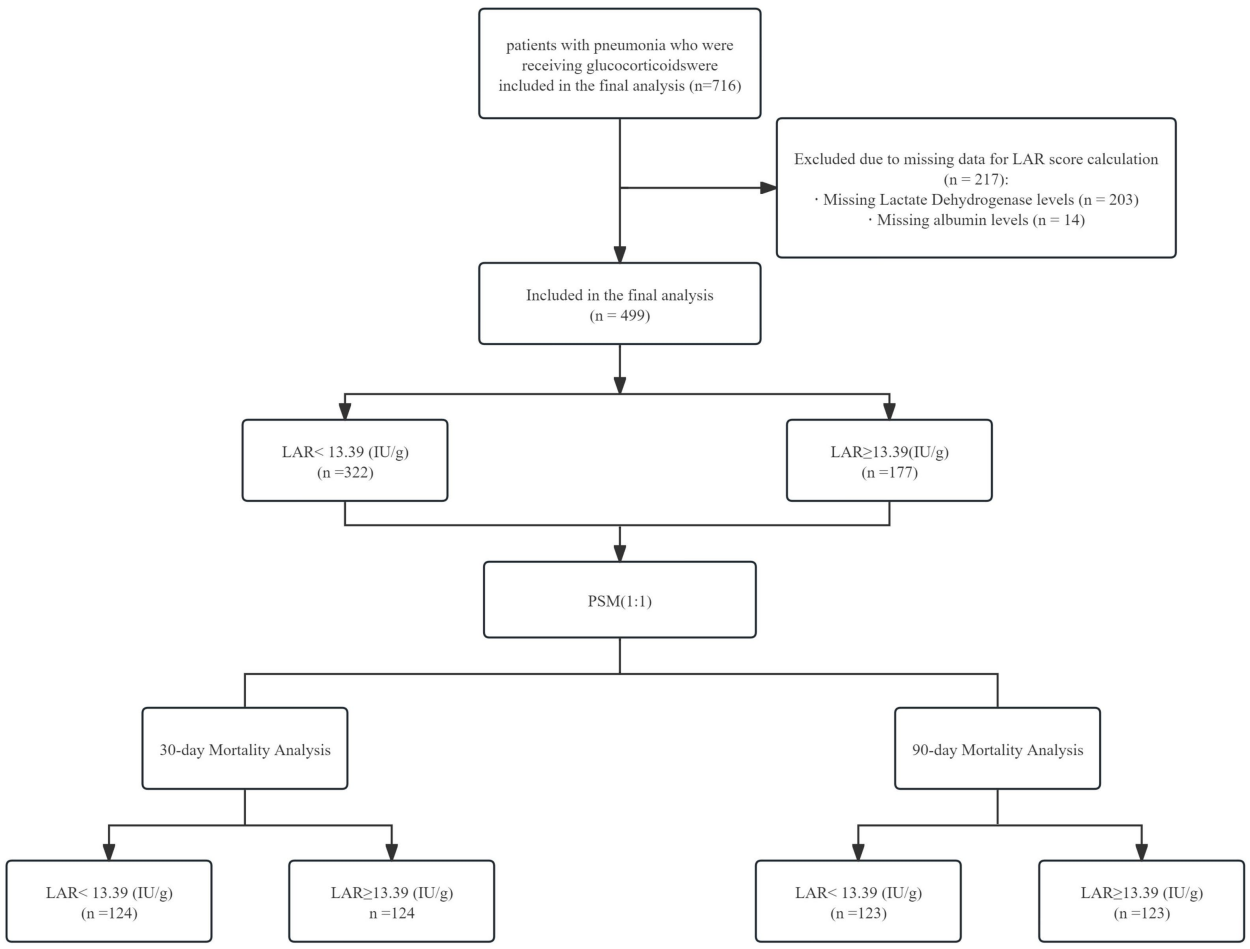
Flowchart of the study cohort.

Kaplan–Meier curves in the matched cohort showed lower survival probabilities in the high-LAR group for both endpoints (Figure 4). The curves diverged early and remained separated throughout follow-up (30-day log-rank *p* = 0.042; 90-day *p* = 0.032), highlighting the ability of LAR to distinguish survival trajectories independent of baseline covariates. Subgroup analyses confirmed that the association between high LAR and increased mortality was consistent across strata of age, sex, CURB-65 score, high-dose glucocorticoid use, and mechanical ventilation (all interaction *p* > 0.05) (Figure 5) with a statistically significant interaction observed only for diabetes mellitus in the 90-day mortality analysis (*p* = 0.034) (Supplementary Figure S5).

### Sensitivity analyses

A series of sensitivity analyses conducted in the unmatched original cohort further corroborated the robustness of our findings. First, when LAR was modeled as a continuous variable in multivariable Cox regression, each 1-unit increase in LAR was associated with a 2% increase in the risk of 30-day mortality (adjusted HR = 1.02, 95% CI: 1.01–1.03, *p* = 0.002), with a similar magnitude of increased risk observed for 90-day mortality (*p* = 0.006) (Supplementary Table S5). Second, trend testing across LAR tertiles clearly demonstrated a dose–response gradient: the risk of mortality increased incrementally with higher LAR tertile levels (trend test *p* < 0.01) (Supplementary Table S5). Furthermore, repeating the multivariable Cox regression analysis in the original cohort using the same binary cut-off yielded effect estimates that were consistent in direction and comparable in magnitude to the doubly robust estimates from the matched cohort. Kaplan–Meier curves in the original cohort also showed early and sustained separation between groups (log-rank *p* < 0.0001) (Supplementary Figure S2). Subgroup analyses in the original cohort yielded results consistent with those from the matched cohort, with a statistically significant interaction again observed only for diabetes mellitus in the 90-day mortality analysis (Supplementary Figure S3). The complete-case analysis produced results that were highly consistent with the primary analysis (Supplementary Table S6, Supplementary Figure S4).

## Discussion

In this secondary analysis of 499 ICU patients with pneumonia receiving glucocorticoid therapy, an elevated lactate dehydrogenase-to-albumin ratio (LAR) emerged as a robust and independent predictor of 30-day and 90-day all-cause mortality. To our knowledge, this represents the first investigation to validate a steroid-resilient prognostic biomarker specifically within this therapeutically confounded population. A single admission LAR measurement consistently stratified patients into distinct risk categories across multiple analytical frameworks, including multivariable adjustment, propensity score matching, and doubly robust estimation (24).

The prognostic utility of LAR in this setting likely reflects its capacity to integrate signals of both irreversible cellular injury and diminishing physiological reserve—two pathophysiological hallmarks of severe pneumonia that are largely unaffected by the genomic actions of glucocorticoids. Unlike C-reactive protein, whose hepatic synthesis is directly suppressed by steroids (10), lactate dehydrogenase is released upon cell membrane disruption driven by pathogen-induced cytolysis and hypoxic pyroptosis, processes minimally influenced by exogenous corticosteroids (14). Albumin complements this by capturing hepatic synthetic function, capillary leakage, and endothelial dysfunction (11–13). The blood urea nitrogen-to-albumin ratio (BUN/ALB), another composite marker reflecting prerenal azotemia and catabolic stress, has also demonstrated prognostic value in pneumonia (16, 17), including in glucocorticoid-treated subgroups (33). This convergence of evidence underscores the clinical relevance of “metabolic-injury” composite indices in this vulnerable population. By combining these mechanistically distinct domains, LAR may provide a more faithful representation of overall disease burden than either component alone.

Baseline characteristics reinforced a “high-injury, low-reserve” phenotype in the high-LAR group, which exhibited markedly elevated LDH, reduced albumin, and greater requirements for mechanical ventilation and vasoactive support. Notably, despite greater illness severity, these patients received lower daily glucocorticoid doses—an observation that may reflect confounding by indication, wherein clinicians cautiously limited steroid exposure in the most critically ill individuals due to concerns about secondary infections, or alternatively, a phenotype of relative corticosteroid hyporesponsiveness (2, 34, 35). This underscores that mortality in this cohort was predominantly driven by the underlying disease burden rather than by steroid exposure per se. Recent meta-analyses indicate that low-dose, short-course glucocorticoid regimens confer a survival benefit in severe pneumonia (approximately 18–27% relative risk reduction), with no significant increase in serious adverse events (36, 37), yet cumulative doses exceeding 20 g of methylprednisolone equivalent may elevate mortality risk (28). These findings collectively highlight the need for nuanced, individualized risk–benefit assessment in this population.

The statistical findings aligned with this biological rationale. LAR demonstrated discriminative performance comparable to the more complex Pneumonia Severity Index and outperformed CURB-65 and LDH alone. The association between high LAR and mortality remained consistent across nine analytical frameworks, with hazard ratios ranging from 1.56 to 2.55 in fully adjusted and doubly robust models. Importantly, the independent prognostic value of LAR persisted irrespective of whether PSI or CURB-65 was used as the severity-adjustment variable, mitigating concerns about selective reporting.

From a translational perspective, LAR offers practical advantages as a low-cost, rapidly available biomarker. Its high negative predictive value (87.9% for 30-day mortality) suggests utility in identifying lower-risk patients who may not require immediate escalation of care. Conversely, an admission LAR ≥ 13.39 could prompt intensified monitoring—for instance, serial assessments every 48–72 h—and earlier consideration of organ support strategies. In long-term glucocorticoid users hospitalized with intercurrent infection, LAR may serve as an objective baseline risk indicator. These preliminary considerations require prospective validation.

Several limitations warrant acknowledgment. The observational, secondary nature of the analysis precludes full adjustment for glucocorticoid type, precise dosing, and treatment duration. Residual confounding cannot be entirely excluded. LAR was measured only at ICU admission, and serial measurements—potentially informative for dynamic risk assessment—were unavailable. The exclusively East Asian cohort limits generalizability to other populations. Finally, the role of specific LDH isoenzymes in immune-metabolic pathways merits further mechanistic investigation (38).

## Conclusion

In conclusion, the lactate dehydrogenase-to-albumin ratio is a simple, inexpensive, and powerful steroid-resilient prognostic biomarker for mortality in pneumonia patients receiving glucocorticoids. By piercing through the confounding veil of steroid-induced signal distortion, LAR offers clinicians a practical tool to identify high-risk individuals who warrant escalated monitoring and targeted intervention. Further prospective multicenter studies are warranted to validate these findings and explore the utility of dynamic LAR monitoring in guiding clinical decision-making and optimizing patient outcomes.

## Data Availability

Publicly available datasets were analyzed in this study. This data can be found at: Data are available in a public, open access repository. Extra data can be accessed via the Dryad data repository at https://datadryad.org/stashe with the doi: 10.5061/dryad.mkkwh70x2.

## Glossary

ALB: Serum Albumin
ARDS: Acute Respiratory Distress Syndrome
ATS/IDSA: American Thoracic Society/Infectious Diseases Society of America
AUC: Area Under the Curve
BUN: Blood Urea Nitrogen
CHD: Coronary Heart Disease
CI: Confidence Interval
CRE: Creatinine
CRP: C-Reactive Protein
DM: Diabetes Mellitus
HGB: Hemoglobin
HR: Hazard Ratio
ICU: Intensive Care Unit
IDI: Integrated Discrimination Improvement
IPTW: Inverse Probability of Treatment Weighting
IQR: Interquartile Range
LAR: Lactate Dehydrogenase-to-Albumin Ratio
LDH: Lactate Dehydrogenase
MICE: Multiple Imputation by Chained Equations
NRI: Net Reclassification Improvement
OW: Overlap Weighting
PCT: Procalcitonin
PH: Proportional Hazards
PSI: Pneumonia Severity Index
PSM: Propensity Score Matching
ROC: Receiver Operating Characteristic
SD: Standard Deviation
SMD: Standardized Mean Difference
STROBE: Strengthening the Reporting of Observational Studies in Epidemiology
WBC: White Blood Cell Count
