# Supplementary material for: Lactate dehydrogenase-to-albumin ratio predicts 30-day and 90-day mortality in glucocorticoid-treated ICU patients with pneumonia: a secondary analysis of a multicenter cohort
Source: Front Med (Lausanne). 2026 May 5;13:1832943. doi: 10.3389/fmed.2026.1832943 (PMC13183611; doi:10.3389/fmed.2026.1832943)
Supplement: Supplementary file 1 [file Data_Sheet_1.PDF]

Supplementary Table S1 The variable distribution of the missing data

Variable	Miss.freq	Missing Percentage (%)
Age	0	0
Antibiotics	0	0
BUN	1	0.20
CHD	0	0
Cirrhosis	0	0
CRE	2	0.40
DM	0	0
Gender	0	0
Glucocorticoid accumulation	66	13.22
HGB	3	0.60
PSI	0	0
Vasoactive drugs	0	0
Ventilation	0	0
WBC	3	0.60

Abbreviations: BUN, blood urea nitrogen; CHD, coronary heart disease; CRE, creatinine; DM, diabetes mellitus; HGB, hemoglobin; PSI, pneumonia severity index; WBC, white blood cell count.

A

B

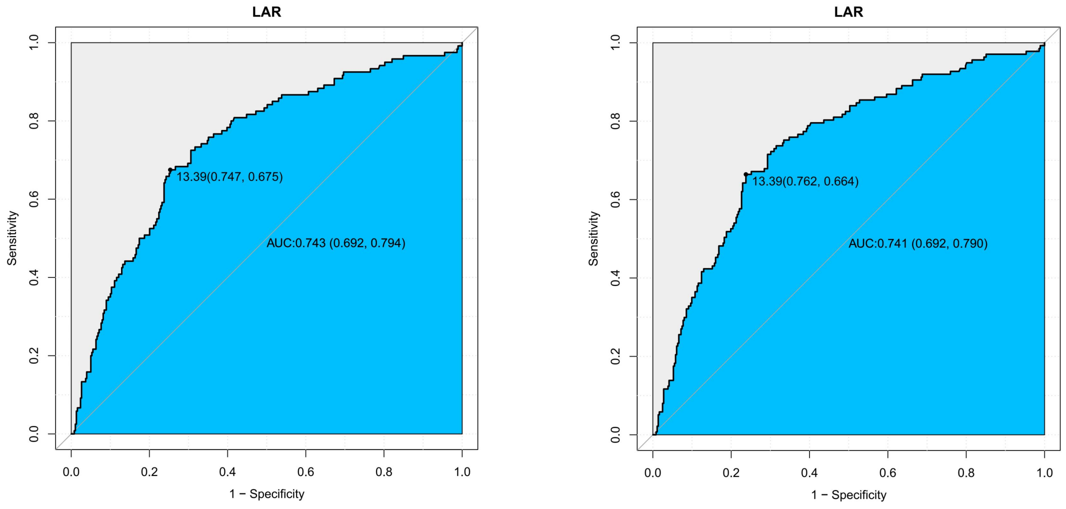

Supplementary Figure S1. Receiver Operating Characteristic (ROC) Curves of LAR for Predicting 30-Day and 90-Day Mortality in the Original Cohort.

(A) ROC curve for 30-day mortality with an AUC of 0.743 (95% CI: 0.692–0.794); (B) ROC curve for 90-day mortality with an AUC of 0.741 (95% CI: 0.692–0.790). The blue shaded area represents the area under the curve. The optimal LAR cutoff value of 13.39 was identified using the maximum Youden Index, corresponding to a sensitivity of 67.5% and specificity of 74.7% for 30-day mortality, and a sensitivity of 66.4% and specificity of 76.2% for 90-day mortality. The coordinates marked on the curves (0.747, 0.675) and (0.762, 0.664) represent the points on the ROC curve at the optimal threshold, where the x-coordinate indicates 1-Specificity and the y-coordinate indicates Sensitivity. Detailed diagnostic performance metrics are summarized in Supplementary Table S2.

Abbreviations: AUC, area under the curve; LAR, lactate dehydrogenase-to-albumin ratio.

Supplementary Table S2. Predictive performance of LAR for 30-day and 90-day mortality in the pre-matching cohort.

Diagnostic Metric	30-day Mortality	90-day Mortality
Optimal Threshold (Cut-off)	13.39	13.39
Area Under the Curve (AUC)	0.743 (0.692–0.794)	0.741 (0.692–0.790)
Sensitivity (%)	67.5	66.4
Specificity (%)	74.7	76.2
Positive Predictive Value (PPV, %)	45.8	51.4
Negative Predictive Value (NPV, %)	87.9	85.7
Overall Accuracy (%)	73.0	73.6
Youden Index	0.42	0.43
True Positive (TP) / True Negative (TN)	81 / 283	91 / 276
False Positive (FP) / False Negative (FN)	96 / 39	86 / 46

Notes: Data in parentheses for AUC represent 95% confidence intervals.

Abbreviations: FN, false negative; FP, false positive; LAR, lactate dehydrogenase-to-albumin ratio; NPV, negative predictive value; PPV, positive predictive value; TN, true negative; TP, true positive.

Supplementary Table S3. Baseline Characteristics Before and After Propensity Score Matching (30-day Mortality Analysis)

Variables	Total n = 499 (100%)	Primary cohort			PSM cohort		
		Low-LAR Ratio n = 322(64.5%)	High-LAR Ratio n = 177(35.5%)	***P* value**	Low-LAR Ratio n = 124(50%)	High-LAR Ratio n = 124(50%)	***P* value**
**Age**[c] ,years				0.055			0.611
＜60	239 (47.9)	144 (44.7)	95 (53.7)		61 (49.2)	65 (52.4)	
≥60	260 (52.1)	178 (55.3)	82 (46.3)		63 (50.8)	59 (47.6)	
**Female**[c]	245 (49.1)	159 (49.4)	86 (48.6)	0.866	53 (42.7)	65 (52.4)	0.127
**Ventilation**[c]	182 (36.5)	66 (20.5)	116 (65.5)	< 0.001	61 (49.2)	63 (50.8)	0.799
**PSI**[a], points	80.3 ± 31.3	75.8 ± 29.5	88.5 ± 32.8	< 0.001	83.6 ± 33.4	82.5 ± 31.3	0.79
**Vasoactive drugs**[c]	85 (17.0)	29 (9)	56 (31.6)	< 0.001	25 (20.2)	17 (13.7)	0.176
**Glucocorticoid accumulation**[b], mg	4.3 (2.3, 9.6)	5.4 (2.5, 12.3)	3.3 (1.9, 5.8)	< 0.001	4.1 (2.4, 7.8)	4.1 (2.2, 6.8)	0.812
**HGB**[a], g/L	113.0 ± 23.0	114.5 ± 23.5	110.1 ± 21.9	0.042	113.2 ± 21.9	111.4 ± 21.6	0.515
**BUN**[b], mmol/L	6.1 (4.6, 9.1)	5.7 (4.3, 8.1)	7.1 (5.3, 12.0)	< 0.001	6.0 (4.4, 9.6)	6.3 (5.0, 9.8)	0.323
**CHD**[c]	58 (11.6)	37 (11.5)	21 (11.9)	0.901	14 (11.3)	13 (10.5)	0.838
**Cirrhosis**[c]	4 (0.8)	2 (0.6)	2 (1.1)	0.618	2 (1.6)	1 (0.8)	1
**DM**[c]	127 (25.5)	87 (27)	40 (22.6)	0.278	33 (26.6)	31 (25)	0.772
**WBC**[a], $\times 10^9$/L	7.8 (5.6, 11.3)	7.5 (5.5, 10.7)	8.9 (6.2, 12.1)	0.002	7.6 (5.5, 11.1)	8.5 (6.2, 11.9)	0.124
**Antibiotics**[c]	339 (67.9)	204 (63.4)	135 (76.3)	0.003	88 (71)	87 (70.2)	0.889
**CRE**[b], μmol/L	63.7 (50.2, 88.3)	63.2 (51.3, 82.2)	65.0 (48.3, 105.0)	0.405	61.8 (49.2, 88.5)	60.5 (46.6, 89.3)	0.948
**30-day mortality**[c]	120 (24.0)	39 (12.1)	81 (45.8)	< 0.001	33 (26.6)	46 (37.1)	0.076

Notes: Data presented are mean ± SD[a], median (IQR)[b], or N (%)[c].

Abbreviations:PSI, pneumonia severity index; HGB, hemoglobin; BUN, blood urea nitrogen; CHD, coronary heart disease; DM, diabetes mellitus; WBC, white blood cell count; LAR,lactate dehydrogenase to albumin ratio; CRE, creatinine; SD, standard deviation; IQR, interquartile range.

Supplementary Table S4. Baseline Characteristics Before and After Propensity Score Matching (90-day Mortality Analysis)

Variables	Total n = 499 (100%)	Primary cohort Low-LAR Ratio n = 322(64.5%)	Primary cohort High-LAR Ratio n = 177(35.5%)	Primary cohort ***P* value**	PSM cohort Low-LAR Ratio n = 123(50%)	PSM cohort High-LAR Ratio n = 123(50%)	PSM cohort ***P* value**
**Age**[c] ,years				0.055			0.444
＜60	239 (47.9)	144 (44.7)	95 (53.7)		59 (48)	65 (52.8)	
≥60	260 (52.1)	178 (55.3)	82 (46.3)		64 (52)	58 (47.2)	
**Female**[c]	245 (49.1)	159 (49.4)	86 (48.6)	0.866	61 (49.6)	65 (52.8)	0.61
**Ventilation**[c]	182 (36.5)	66 (20.5)	116 (65.5)	< 0.001	59 (48)	62 (50.4)	0.702
**PSI**[a], points	80.3 ± 31.3	75.8 ± 29.5	88.5 ± 32.8	< 0.001	84.8 ± 34.8	82.4 ± 31.4	0.565
**Vasoactive drugs**[c]	85 (17.0)	29 (9)	56 (31.6)	< 0.001	25 (20.3)	18 (14.6)	0.24
**Glucocorticoid accumulation**[b],mg	4.3 (2.2, 9.1)	5.3 (2.4, 11.9)	3.2 (1.9, 5.5)	< 0.001	3.6 (2.0, 6.9)	3.7 (2.2, 6.2)	0.892
**HGB**[a], g/L	113.0 ± 23.0	114.6 ± 23.5	110.1 ± 21.9	0.039	111.5 ± 25.3	111.4 ± 21.6	0.974
**BUN**[b], mmol/L	6.1 (4.6, 9.1)	5.7 (4.3, 8.1)	7.1 (5.3, 12.0)	< 0.001	6.6 (4.7, 10.7)	6.3 (5.0, 9.8)	0.829
**CHD**[c]	58 (11.6)	37 (11.5)	21 (11.9)	0.901	15 (12.2)	13 (10.6)	0.688
**Cirrhosis**[c]	4 (0.8)	2 (0.6)	2 (1.1)	0.618	2 (1.6)	1 (0.8)	1
**DM**[c]	127 (25.5)	87 (27)	40 (22.6)	0.278	32 (26)	30 (24.4)	0.769
**WBC**[a], $\times 10^9$/L	7.8 (5.6, 11.3)	7.5 (5.5, 10.7)	8.9 (6.2, 12.1)	0.001	7.8 (5.8, 11.3)	8.6 (6.2, 11.9)	0.409
**Antibiotics**[c]	339 (67.9)	204 (63.4)	135 (76.3)	0.003	88 (71.5)	86 (69.9)	0.779
**CRE**[b], μmol/L	63.7 (50.2, 88.3)	63.2 (51.3, 82.2)	66.9 (48.3, 105.0)	0.371	64.0 (51.8, 93.7)	61.9 (46.6, 89.6)	0.371
**90-day mortality**[c]	137 (27.5)	46 (14.3)	91 (51.4)	< 0.001	36 (29.3)	50 (40.7)	0.061

Notes: Data presented are mean ± SD[a], median (IQR)[b], or N (%)[c].

Abbreviations:PSI, pneumonia severity index; HGB, hemoglobin; BUN, blood urea nitrogen; CHD, coronary heart disease; DM, diabetes mellitus; WBC, white blood cell count; LAR,lactate dehydrogenase to albumin ratio; CRE, creatinine; SD, standard deviation; IQR, interquartile range.

Supplementary Table S5 Multivariate Cox Regression Analysis of the Association Between LAR Ratio and In-Hospital Mortality in Pneumonia Patients Treated with Glucocorticoids in the original cohort.

Variable	Crude Model HR (95%CI)	Crude Model *P* value	Model 1 HR (95%CI)	Model 1 *P* value	Model 2 HR (95%CI)	Model 2 *P* value	Model 3 HR (95%CI)	Model 3 *P* value	Model 4 HR (95%CI)	Model 4 *P* value
**30-mortality**										
**LAR (per 1-unit increase)**	1.02 (1.01~1.02)	<0.001	1.02 (1.01~1.02)	<0.001	1.02 (1.01~1.02)	<0.001	1.02(1.01~1.03)	0.002	1.01 (1~1.03)	0.014
**LAR ＜13.39**	1(Ref)		1(Ref)		1(Ref)		1(Ref)		1(Ref)	
**LAR ≥13.39**	4.9 (3.34~7.19)	<0.001	5.05 (3.44~7.42)	<0.001	5.09 (3.47~7.48)	<0.001	2.07 (1.34~3.2)	0.001	2.18 (1.4~3.4)	0.001
**Quantile**										
**LAR ＜10.25**	1(Ref)		1(Ref)		1(Ref)		1(Ref)		1(Ref)	
**LAR ≥10.25**	4.55 (2.93~7.07)	<0.001	4.63 (2.98~7.21)	<0.001	4.75 (3.05~7.38)	<0.001	1.87 (1.15~3.05)	0.011	1.91 (1.16~3.14)	0.011
**Quantile**										
**LAR< 7.892**	1(Ref)		1(Ref)		1(Ref)		1(Ref)		1(Ref)	
**LAR 7.892-13.951**	1.75 (0.94~3.25)	0.076	1.73 (0.93~3.21)	0.082	1.76 (0.95~3.26)	0.074	1.16 (0.61~2.22)	0.646	1 (0.51~1.93)	0.991
**LAR＞13.951**	6.24(3.64~10.71)	<0.001	6.42(3.74~11.02)	<0.001	6.52 (3.8~11.2)	<0.001	2.22 (1.22~4.02)	0.009	2.08 (1.13~3.8)	0.018
Trend test		<0.001		<0.001		<0.001		0.002		0.003
**90-mortality**										
**LAR (per 1-unit increase)**	1.02 (1.01~1.02)	<0.001	1.02 (1.01~1.02)	<0.001	1.02 (1.01~1.02)	<0.001	1.01 (1~1.02)	0.006	1.01 (1~1.02)	0.032
**LAR<13.3882**	1(Ref)		1(Ref)		1(Ref)		1(Ref)		1(Ref)	
**LAR≥13.3882**	4.82 (3.38~6.87)	<0.001	5.03 (3.52~7.19)	<0.001	5.08 (3.55~7.26)	<0.001	1.93(1.29~2.89)	0.001	2.02 (1.34~3.05)	0.001
**Quantile**										
**LAR<10.25**	1(Ref)		1(Ref)		1(Ref)		1(Ref)		1(Ref)	
**LAR≥10.25**	4.39 (2.93~6.58)	<0.001	4.51 (3.01~6.77)	<0.001	4.63 (3.08~6.95)	<0.001	1.78 (1.13~2.78)	0.012	1.8 (1.13~2.84)	0.013
**Quantile**										
**LAR< 7.892**	1(Ref)		1(Ref)		1(Ref)		1(Ref)		1(Ref)	
**LAR 7.892-13.951**	1.81 (1.03~3.19)	0.039	1.79 (1.02~3.16)	0.043	1.82 (1.04~3.2)	0.038	1.25 (0.69~2.25)	0.459	1.08 (0.59~1.99)	0.794
**LAR＞13.951**	6.01 (3.65~9.89)	<0.001	6.25(3.79~10.29)	<0.001	6.35(3.85~10.47)	<0.001	2.06 (1.19~3.56)	0.01	1.92 (1.1~3.36)	0.022
Trend.test		<0.001		<0.001		<0.001		0.004		0.006

Model 1: adjust for Age, Sex

Model 2: adjust for model 1 + CHD, DM and Cirrhosis

Model 3: adjust for model 2 + HGB, WBC, CRE, BUN, Ventilation, Vasoactivedrugs, PSI, Glucocorticoidaccumulation, Antibiotics

Model 4: adjust for model 2 + HGB, WBC, CRE, BUN, Ventilation, Vasoactivedrugs, CURB-65, Glucocorticoidaccumulation, Antibiotics

Abbreviations: BUN, blood urea nitrogen; CHD, coronary heart disease; CRE, creatinine; DM, diabetes mellitus; HGB, hemoglobin; PSI, pneumonia severity index; WBC, white blood cell count; LAR,lactate dehydrogenase to albumin ratio; CURB-65, Confusion, Urea, Respiratory rate, Blood pressure, and Age ≥ 65 years score;

A B

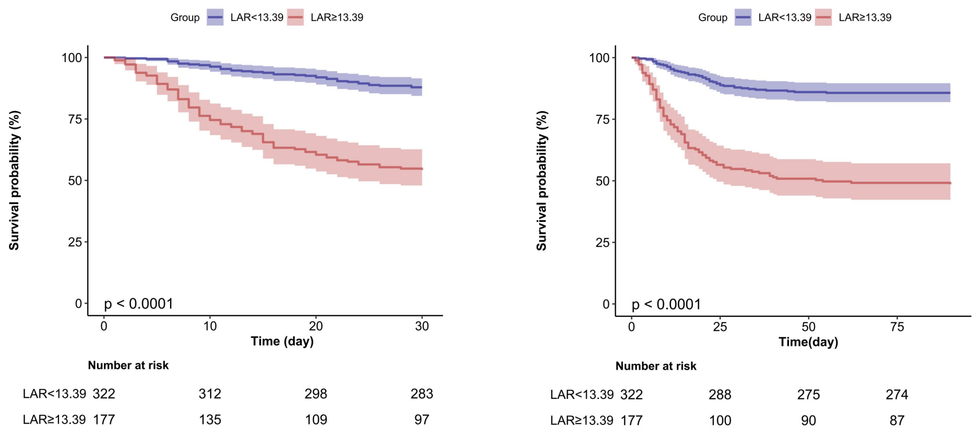

Supplementary Figure S2 Kaplan-Meier Survival Curves Stratified by LAR Levels for 30-Day and 90-Day Mortality in the Original Cohort

Notes:(A) 30-day and (B) 90-day survival curves comparing Low LAR (<13.39, n=322, blue) versus High LAR (≥13.39, n=177, red) groups. Shaded areas represent 95% CIs. Log-rank test: P<0.0001 for both comparisons, indicating significant survival differences between groups. The "Number at risk" tables show patient counts at each follow-up interval.

Abbreviations: CI, confidence interval; LAR, lactate dehydrogenase-to-albumin ratio.

Subgroup	Total	Event (%)1	Event (%)2	HR (95%CI)1	HR (95%CI)2	P for interaction1	P for interaction2
**Overall**							
**Crude**	**499**			**1.016 (1.01~1.022)**	**1.015 (1.009~1.021)**		
**Adjusted**	**499**			**1.016 (1.006~1.026)**	**1.014 (1.004~1.024)**		
**Age**							
<60	239	53 (22.2)	57 (23.8)	1.02 (1~1.04)	1.02 (1~1.04)	0.955	0.871
≥60	260	67 (25.8)	80 (30.8)	1.02 (1~1.03)	1.01 (1~1.03)		
**Sex**							
Male	254	65 (25.6)	75 (29.5)	1.01 (0.99~1.02)	1.01 (0.99~1.02)	0.283	0.319
Female	245	55 (22.4)	62 (25.3)	1.03 (1.01~1.04)	1.02 (1.01~1.04)		
**CURB65**							
≤1	361	67 (18.6)	74 (20.5)	1.02 (1~1.04)	1.02 (1~1.04)	0.471	0.413
>1	138	53 (38.4)	63 (45.7)	1.01 (1~1.03)	1.01 (0.99~1.02)		
**High−dose glucocorticoid**							
No	305	54 (17.7)	62 (20.3)	1.01 (1~1.03)	1.01 (0.99~1.03)	0.808	0.704
Yes	194	66 (34)	75 (38.7)	1.02 (1~1.04)	1.02 (1~1.03)		
**Ventilation**							
No	317	19 (6)	20 (6.3)	1.01 (0.99~1.04)	1.01 (0.99~1.03)	0.777	0.838
Yes	182	101 (55.5)	117 (64.3)	1.02 (1~1.03)	1.02 (1~1.03)		
**DM**							
No	372	90 (24.2)	100 (26.9)	1.01 (1~1.02)	1.01 (1~1.02)	0.077	0.034
Yes	127	30 (23.6)	37 (29.1)	1.06 (1.01~1.11)	1.06 (1.02~1.11)		

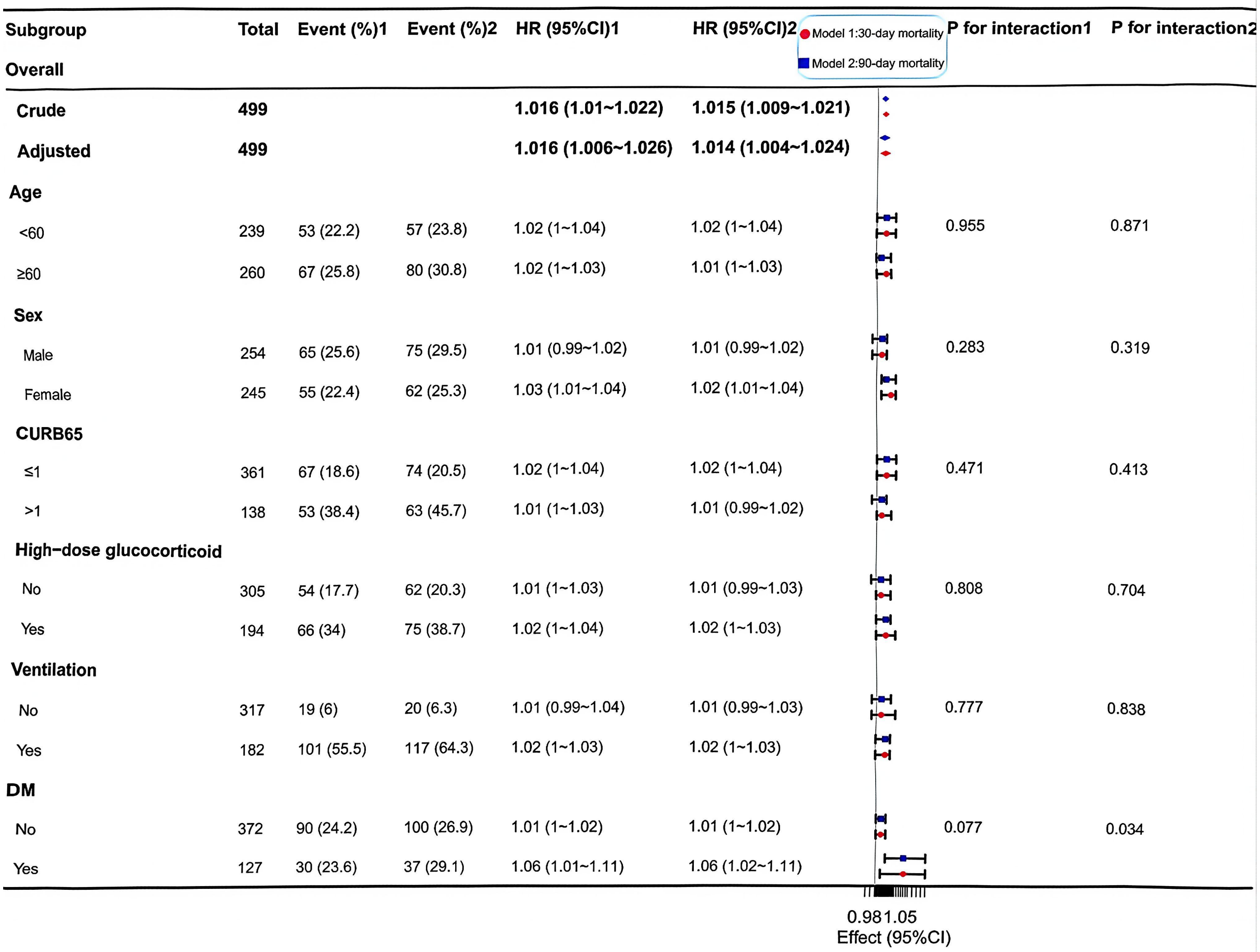

Supplementary Figure S3. Forest Plot of Hazard Ratios for LAR-Associated Mortality Risk Across Clinical Subgroups

Notes: Forest plot showing HRs (95% CIs) for 30-day (red) and 90-day (blue) mortality per 1-unit increase in LAR. Overall analysis includes both crude and adjusted estimates from the PSM cohort. Subgroup stratification by age, sex, CURB-65 score, glucocorticoid use, mechanical ventilation, and diabetes mellitus. A significant interaction was observed for DM in 90-day mortality (P = 0.034), indicating enhanced LAR-associated mortality risk in diabetic patients.

Abbreviations: CI, confidence interval; CURB-65, Confusion, Urea, Respiratory rate, Blood pressure, and Age ≥65 years score; DM, diabetes mellitus; HR, hazard ratio; LAR, lactate dehydrogenase-to-albumin ratio; PSM, propensity score matching.

Supplementary Table S6 Multivariate Cox Regression Analysis (Complete Case Analysis) of the Association Between LAR Ratio and In-Hospital Mortality in Pneumonia Patients Treated with Glucocorticoids.

Variable	Crude Model		Model 1		Model 2		Model 3		Model 4	
	HR (95%CI)	*P* value	HR (95%CI)	*P* value	HR (95%CI)	*P* value	HR (95%CI)	*P* value	HR (95%CI)	*P* value
**30-mortality**										
**LAR （per 1-unit increase)**	1.02 (1.02~1.03)	<0.001	1.03 (1.02~1.04)	<0.001	1.03 (1.02~1.04)	<0.001	1.02 (1.01~1.04)	0.001	1.02 (1~1.03)	0.01
**LAR<13.39**	1(Ref)		1(Ref)		1(Ref)		1(Ref)		1(Ref)	
**LAR ≥13.39**	4.48 (3~6.7)	<0.001	4.66 (3.1~6.98)	<0.001	4.7 (3.13~7.05)	<0.001	2.06 (1.3~3.27)	0.002	2.24 (1.4~3.6)	0.001
**Quantile**										
**LAR<10.44**	1(Ref)		1(Ref)		1(Ref)		1(Ref)		1(Ref)	
**LAR ≥10.44**	4.13 (2.62~6.51)	<0.001	4.22 (2.67~6.65)	<0.001	4.28 (2.71~6.75)	<0.001	1.9 (1.15~3.14)	0.013	1.98 (1.18~3.34)	0.01
**Quantile**										
**LAR< 8.036**	1(Ref)		1(Ref)		1(Ref)		1(Ref)		1(Ref)	
**LAR 8.036-14.238**	2.16 (1.14~4.1)	0.018	2.13 (1.13~4.04)	0.02	2.14 (1.13~4.06)	0.019	1.48 (0.76~2.87)	0.251	1.27 (0.64~2.52)	0.495
**LAR＞14.238**	5.96 (3.34~10.63)	<0.001	6.19 (3.46~11.08)	<0.001	6.26 (3.5~11.22)	<0.001	2.38 (1.25~4.52)	0.008	2.32 (1.21~4.46)	0.011
Trend test		<0.001		<0.001		<0.001		0.004		0.004
**90-mortality**										
**LAR （per 1-unit increase)**	1.02 (1.02~1.03)	<0.001	1.03 (1.02~1.03)	<0.001	1.03 (1.02~1.03)	<0.001	1.02 (1.01~1.03)	0.003	1.02 (1~1.03)	0.019
**LAR<13.39**	1(Ref)		1(Ref)		1(Ref)		1(Ref)		1(Ref)	
**LAR≥13.39**	4.39 (3.02~6.39)	<0.001	4.62 (3.16~6.74)	<0.001	4.68 (3.2~6.85)	<0.001	1.9 (1.24~2.92)	0.003	2.05 (1.32~3.19)	0.001

Variable	Crude Model HR (95%CI)	Crude Model *P* value	Model 1 HR (95%CI)	Model 1 *P* value	Model 2 HR (95%CI)	Model 2 *P* value	Model 3 HR (95%CI)	Model 3 *P* value	Model 4 HR (95%CI)	Model 4 *P* value
**Quantile**										
**LAR<10.44**	1(Ref)		1(Ref)		1(Ref)		1(Ref)		1(Ref)	
**LAR ≥10.44**	4.07 (2.66~6.21)	<0.001	4.19 (2.74~6.41)	<0.001	4.27 (2.79~6.54)	<0.001	1.81 (1.13~2.89)	0.013	1.87 (1.15~3.04)	0.012
**Quantile**										
**LAR< 8.036**	1(Ref)		1(Ref)		1(Ref)		1(Ref)		1(Ref)	
**LAR 8.036-14.238**	2.32 (1.28~4.18)	0.005	2.28 (1.26~4.13)	0.006	2.3 (1.27~4.15)	0.006	1.62 (0.87~3.01)	0.125	1.44 (0.76~2.72)	0.261
**LAR＞14.238**	5.87 (3.41~10.11)	<0.001	6.17 (3.57~10.66)	<0.001	6.28 (3.63~10.85)	<0.001	2.23 (1.22~4.07)	0.009	2.19 (1.19~4.05)	0.012
Trend.test		<0.001		<0.001		<0.001		0.008		0.007

Model 1: adjust for Age, Sex

Model 2: adjust for model 1 + CHD, DM and Cirrhosis

Model 3: adjust for model 2 + HGB, WBC, CRE, BUN, Ventilation, Vasoactivedrugs, PSI, Glucocorticoidaccumulation, Antibiotics

Model 4: adjust for model 2 + HGB, WBC, CRE, BUN, Ventilation, Vasoactivedrugs, CURB-65, Glucocorticoidaccumulation, Antibiotics

Abbreviations: BUN, blood urea nitrogen; CHD, coronary heart disease; CRE, creatinine; DM, diabetes mellitus; HGB, hemoglobin; PSI, pneumonia severity index; WBC, white blood cell count; LAR,lactate dehydrogenase to albumin ratio; CURB-65, Confusion, Urea, Respiratory rate, Blood pressure, and Age ≥ 65 years score;

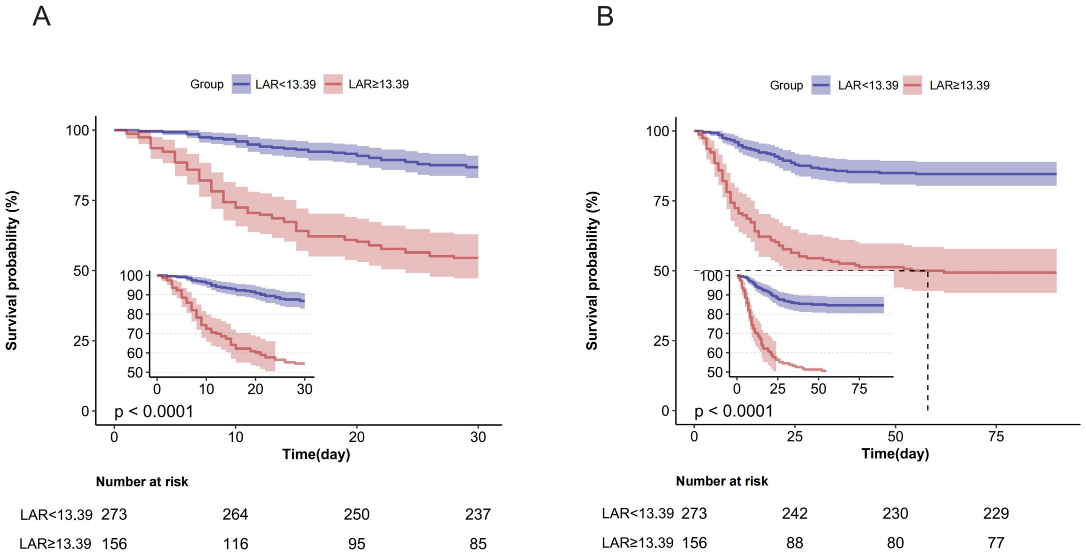

Supplementary Figure S4. Kaplan-Meier Survival Curves Stratified by LAR Levels for 30-Day and 90-Day Mortality in the Complete Case Cohort After Excluding Missing Values

Notes:(A) Kaplan-Meier survival curves for 30-day mortality stratified by the LAR threshold of 13.39. Patients with LAR < 13.39 (blue line) demonstrated significantly higher survival probability compared to those with LAR ≥ 13.39 (red line) (Log-rank test, $p < 0.0001$). Shaded areas represent 95% confidence intervals. The number at risk at different time points is shown below.

(B) Kaplan-Meier survival curves for 90-day mortality stratified by LAR levels in the complete cases. Patients with lower LAR (<13.39) exhibited better survival outcomes than the high LAR group (≥13.39) (Log-rank test, $p < 0.0001$). Confidence intervals are indicated by shaded regions. The corresponding risk table is included.

Abbreviations: CI, confidence interval; LAR, lactate dehydrogenase-to-albumin ratio.
